# Arthroscopy With Adipose-Derived Stromal Vascular Fraction Using a Selective Tissue Engineering Photo-Stimulation Technique for the Treatment of Mild to Moderate Knee Osteoarthritis

**DOI:** 10.1016/j.eats.2024.103015

**Published:** 2024-05-04

**Authors:** José Paulo Gabbi Aramburu Filho, Rafael da Rocha Macedo, Patricio Centurion, Eduardo Branco de Sousa

**Affiliations:** a Orthopaedics and Traumatology Service, Rio de Janeiro Military Police Central Hospital, Rio de Janeiro, Brazil; b Hospital Quinta D’Or, Rio de Janeiro, Brazil; c Hospital IFOR–Rede D’Or São Luiz, São Bernardo do Campo, Brazil; d Discipline of Orthopaedics and Traumatology, ABC Faculty of Medicine, Santo André, Brazil; e Biomedical Sciences Investigation Institute, Ricardo Palma University, Lima, Peru; f General and Specialized Surgery Department, Faculty of Medicine, Fluminense Federal University, Niterói, Brazil

## Abstract

Osteoarthritis (OA) is characterized by articular cartilage degeneration, synovial inflammation, and subchondral bone thickening, affecting the synovial joint as an organ and leading to pain and disability. Subcutaneous stromal vascular fraction is safe and relieves pain, improves function, and repairs cartilage defects in patients with knee OA. Our goal is to describe step-by-step the arthroscopic treatment of mild to moderate knee OA with photo-stimulated stromal vascular fraction harvested from the thigh using a selective tissue engineering photo-stimulation (“One S.T.E.P.”) technique.

Osteoarthritis (OA), which is characterized by articular cartilage degeneration, synovial inflammation, and subchondral bone thickening, affecting the synovial joint as an organ, leads to pain and disability.^1^^,^^2^ It is estimated that over 7% of the world population has OA,^3^ and the knee is one of the most affected joints.^1^^,^^4^ Nonoperative treatment must be the first approach,^5^ whereas total knee replacement has been indicated for patients with severe knee osteoarthritis (KOA) experiencing pain and functional limitations.^6^

Orthobiologics, the use of biologic materials in orthopaedics, has increased in popularity in recent years,^7^ including platelet-rich plasma,^8^^,^^9^ bone marrow aspirate,^10^ bone marrow aspirate concentrate,^10^^,^^11^ mesenchymal stem cells (MSCs), and growth factors. Interest in subcutaneous MSCs, obtained by aspiration, has increased owing to their abundance, easy harvesting with lower donor-site morbidity, and higher progenitor yields than bone marrow aspirate.^12^, ^13^, ^14^

Autologous adipose-derived stromal vascular fraction (SVF), mechanically and/or enzymatically obtained from fat tissue, is safe and relieves pain, improves function, and repairs cartilage defects in patients with KOA.^15^, ^16^, ^17^ Centurion et al. developed the selective tissue engineering photo-stimulation (“One S.T.E.P.”, DMC Equipments, São Paulo, Brazil) technique, with favorable results in preserving adipocytes, besides preserving and stimulating MSCs and their phenotype quality.^18^^,^^19^ Our goal is to describe step-by-step the arthroscopic treatment of mild to moderate KOA with photo-stimulated SVF harvested from the thigh using the One STEP technique.

## Surgical Technique

The procedural steps are summarized in Table 1 and Video 1. First, the patient is anesthetized with spinal anesthesia and is placed in the supine position with the legs in external rotation for harvesting of autologous SVF from the medial thigh. After asepsis and antisepsis of the lower limbs and placement of surgical drapes, the limb to be operated on is prepared with a pneumatic tourniquet positioned on the proximal thigh for the arthroscopic procedure to be performed after SVF harvesting.

**Table 1:** Step-by-Step Guide for Arthroscopy Using Selective Tissue Engineering Photo-Stimulation (One STEP) Technique for Photo-Stimulated SVF Harvesting

1. The patient is anesthetized and placed in the supine position with the right thigh slightly flexed, externally rotated and abducted, and the knee in flexion.
2. The medial thigh is infiltrated with a solution composed of 1,000 mL of saline solution and 1 mL of 1:200,000 epinephrine in the deepest layer of subcutaneous tissue.
3. A 1,210-nm laser is used to dissolve the connective tissue.
4. A liposuction cannula is attached to a 50-mL syringe to obtain about 40 mL of the photo-stimulated subcutaneous tissue.
5. Forty milliliters of the subcutaneous sample is separated into 4 Falcon tubes for centrifugation.
6. The first centrifugation of the subcutaneous solution is performed at 800*g* for 5 min.
7. The infranatant solution is discarded, followed by the second centrifugation at 700*g* for 5 min.
8. The pellet containing the photo-stimulated SVF is aspirated and ready for injection.
9. Knee arthroscopy and joint debridement are performed.
10. SVF injection is performed in the knee joint.

SVF, stromal vascular fraction.

**Fig 1:**
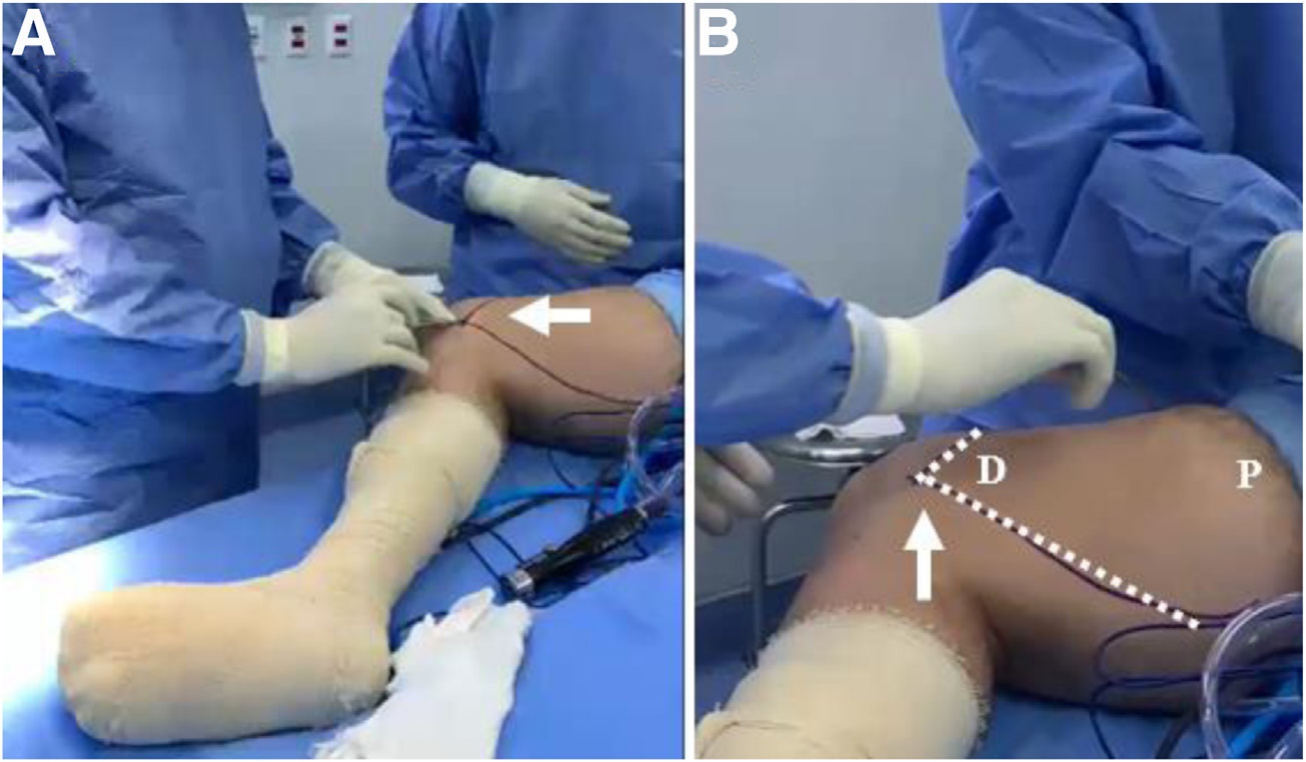
Determination of area for stromal vascular fraction collection. (A) After positioning the patient in the supine position, the right thigh was slightly flexed, externally rotated and abducted, with knee in flexion. The medial epicondyle (arrow) is identified. (B) Two lines (dashed lines) are drawn in the anterolateral and posteromedial direction, representing the anterior and posterior limits for stromal vascular fraction collection. (D, distal thigh; P, proximal thigh; white arrow, medial epicondyle)

Using a dermographic pen, the surgeon draws the limits of the thigh where SVF will be collected. First, the medial epicondyle is identified (Fig 1A). Then, 2 lines are drawn in the anterolateral and posteromedial directions, representing the anterior and posterior limits for safe subcutaneous harvesting (Fig 1B). The point at which both lines meet represents the entrance point for the laser catheter and, then, the aspiration cannula.

The harvesting procedure begins with infiltration of 100 mL of a solution composed of 1,000 mL of saline solution and 1 mL of 1:200,000 epinephrine in the deepest layer of subcutaneous tissue in the ipsilateral inner thigh, which was previously marked. Because this procedure is performed during arthroscopic surgery, there is no need to add lidocaine as in the Klein solution. The surgeon must wait about 15 minutes while the solution takes effect. In the meantime, the material for laser-assisted subcutaneous harvesting is prepared.

**Fig 2:**
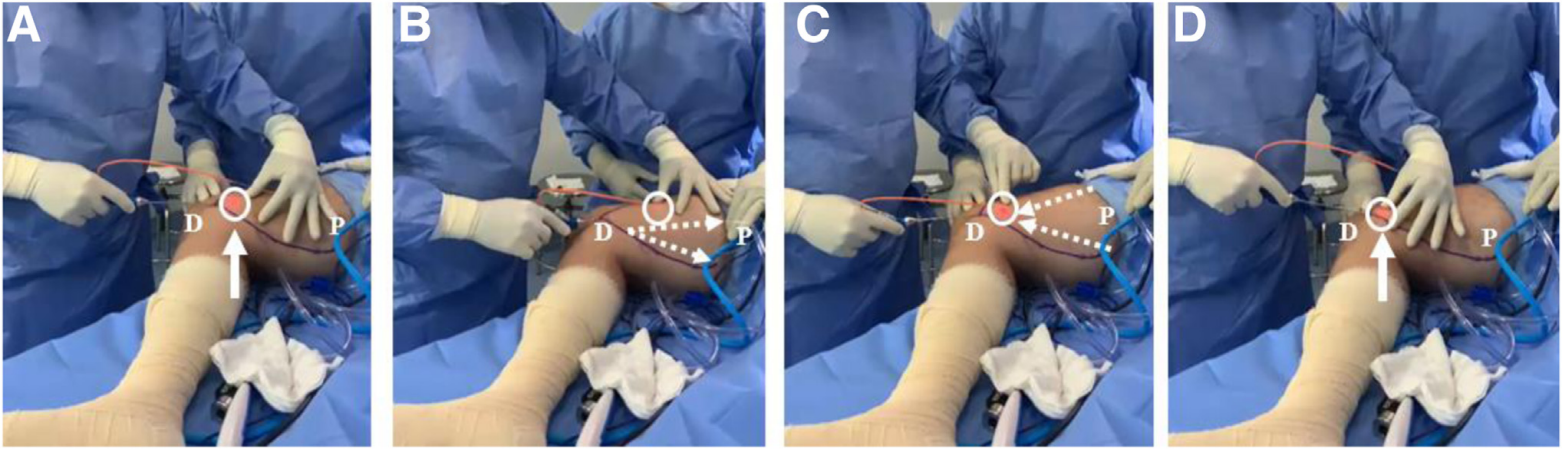
Laser emission using 1,210-nm laser through a 2-mm-diameter catheter to dissolve connective tissue and release parenchyma and stroma (adipocytes and stromal vascular fraction) using photochemical property. (A) The laser catheter is inserted in the medial thigh through the same access positioned at the level of the medial epicondyle (arrow). Forward (B) and backward (C) movements (dotted arrows) must be gently performed, similar to a fan, from the distal thigh (D) to the proximal thigh (P), to loosen the subcutaneous tissue, using the helium-neon red light (circle), positioned in the extremity of the laser catheter, to aid in guidance. (D) Movements must always begin and end in the entrance located at the level of the medial epicondyle (arrow).

A 1,210-nm laser catheter (DMC Equipments, São Paulo, Brazil) is gently inserted into the distal medial thigh subcutaneous tissue through the access positioned at the level of the medial epicondyle (Fig 2A). Forward and backward movements are performed, from distal to proximal, similar to a fan, inside the limits of the anterolateral and posteromedial lines previously drawn to allow the laser to dissolve (denaturalize) the connective tissue and release the parenchyma and stroma (Fig 2 B and C). This procedure must be repeated as many times as needed considering the endpoint as the end of tissue resistance (Fig 2D).

**Fig 3:**
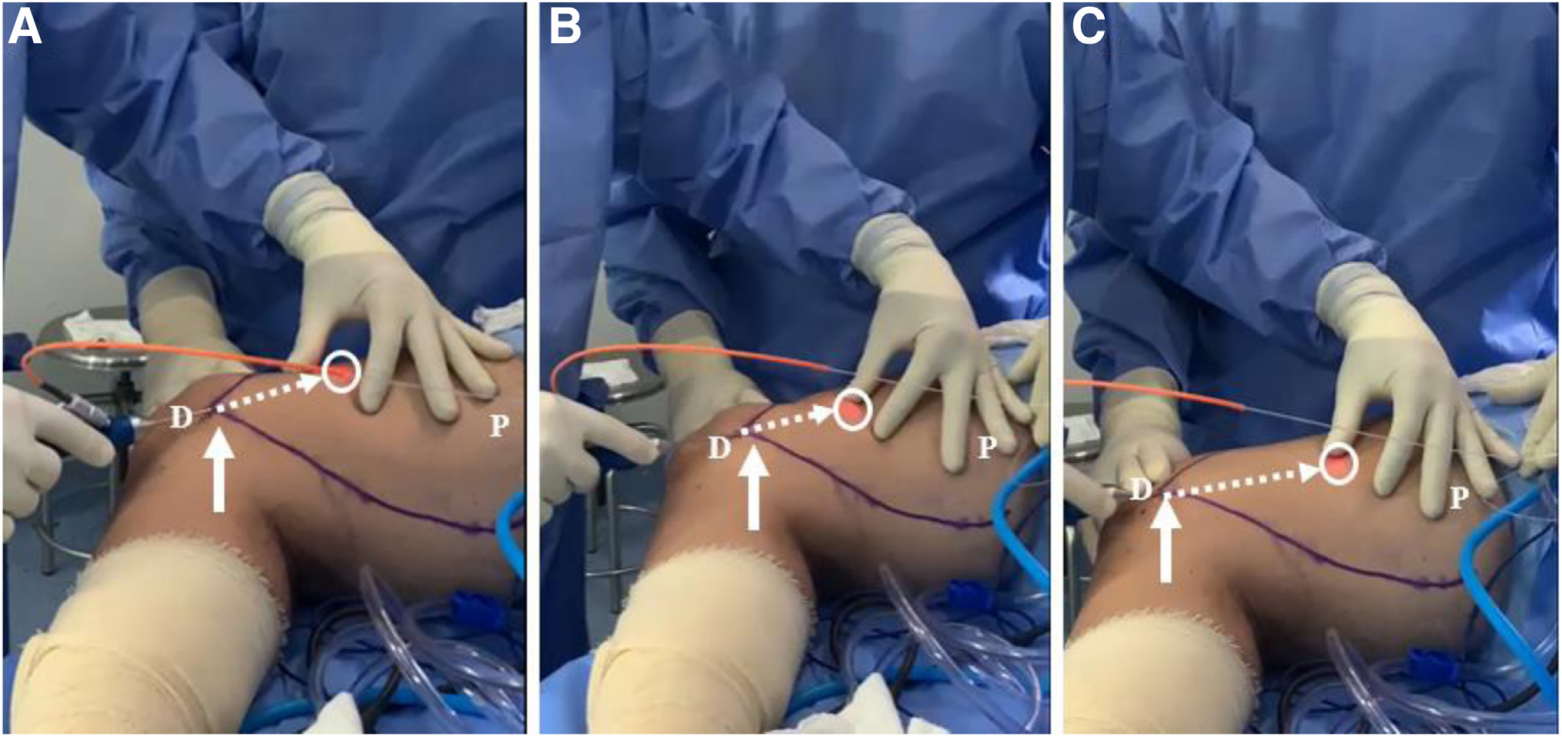
Use of helium-neon red light in extremity of laser catheter to guide connective tissue dissolution. (A) The helium-neon red light positioned in the extremity of the laser catheter (circle) is used to facilitate the guidance of the laser through the subcutaneous tissue. (B, C) One must use the thumb and the index finger to help safely guide the catheter in the right direction, from the distal thigh (D) to the proximal thigh (P) (dotted arrows), without angulating it down to the deeper layers, where the vessels and nerves lie. Movements must always begin and end in the entrance located at the level of the medial epicondyle (solid arrows).

A helium-neon red light positioned in the extremity of the laser catheter facilitates the guidance of the laser through the subcutaneous tissue, indicating the position of the catheter in real time (Fig 3A). The operating room lights should be turned off to help visualize the helium-neon red light. Care must be taken not to change the direction of the catheter into the deeper layers of the thigh, where the vessels and nerves lie. One must use the thumb and the index finger to help guide the catheter, inserted in the medial thigh, from distal to proximal in the safe direction (i.e., in the subcutaneous tissue layer) without angulating it down to the deeper layers (Fig 3 B and C).

**Fig 4:**
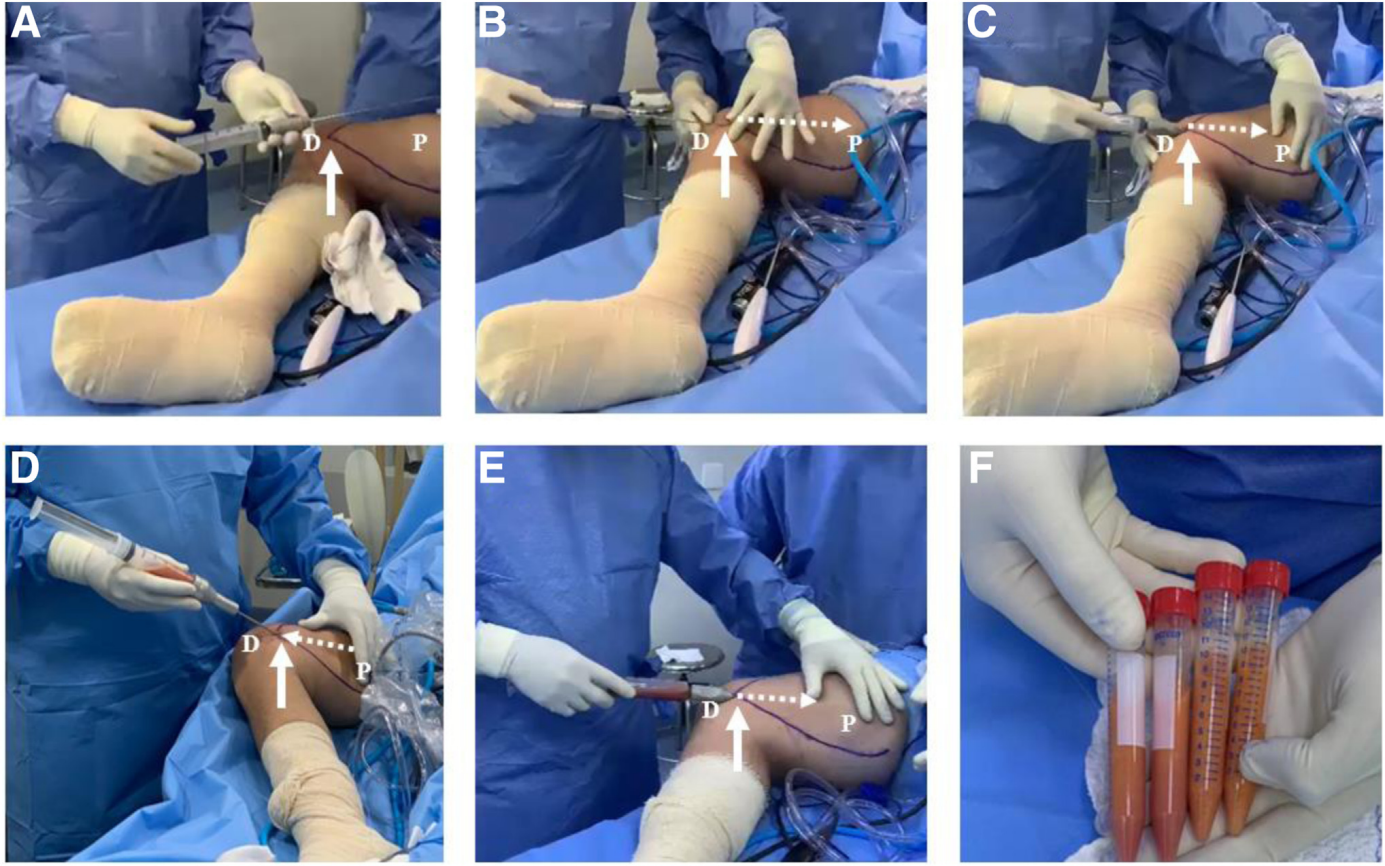
Subcutaneous harvesting. (A) A liposuction cannula is attached to a 50-mL syringe, which is inserted in the medial thigh through the same access positioned at the level of the medial epicondyle (arrow). (B-E) Forward and backward movements (dotted arrows) are performed while pulling back the syringe plunger and using a locking system to maintain the vacuum throughout the harvesting process. Movements must always begin and end in the entrance located at the level of the medial epicondyle (solid arrows). (F) About 40 mL of the photo-stimulated subcutaneous tissue is obtained at the end of the procedure. (D, distal thigh; P, proximal thigh.)

Then, using a liposuction cannula attached to a 50-mL syringe (Fig 4A), pulling back the syringe plunger, and using a locking system to maintain the vacuum throughout the harvesting process (Fig 4B), the surgeon repeats the same forward and backward movements to obtain about 40 mL of subcutaneous tissue at the end of the process (Fig 4C). Care must be taken not to change the direction of the cannula out of the subcutaneous tissue to avoid vascular lesions.

**Fig 5:**
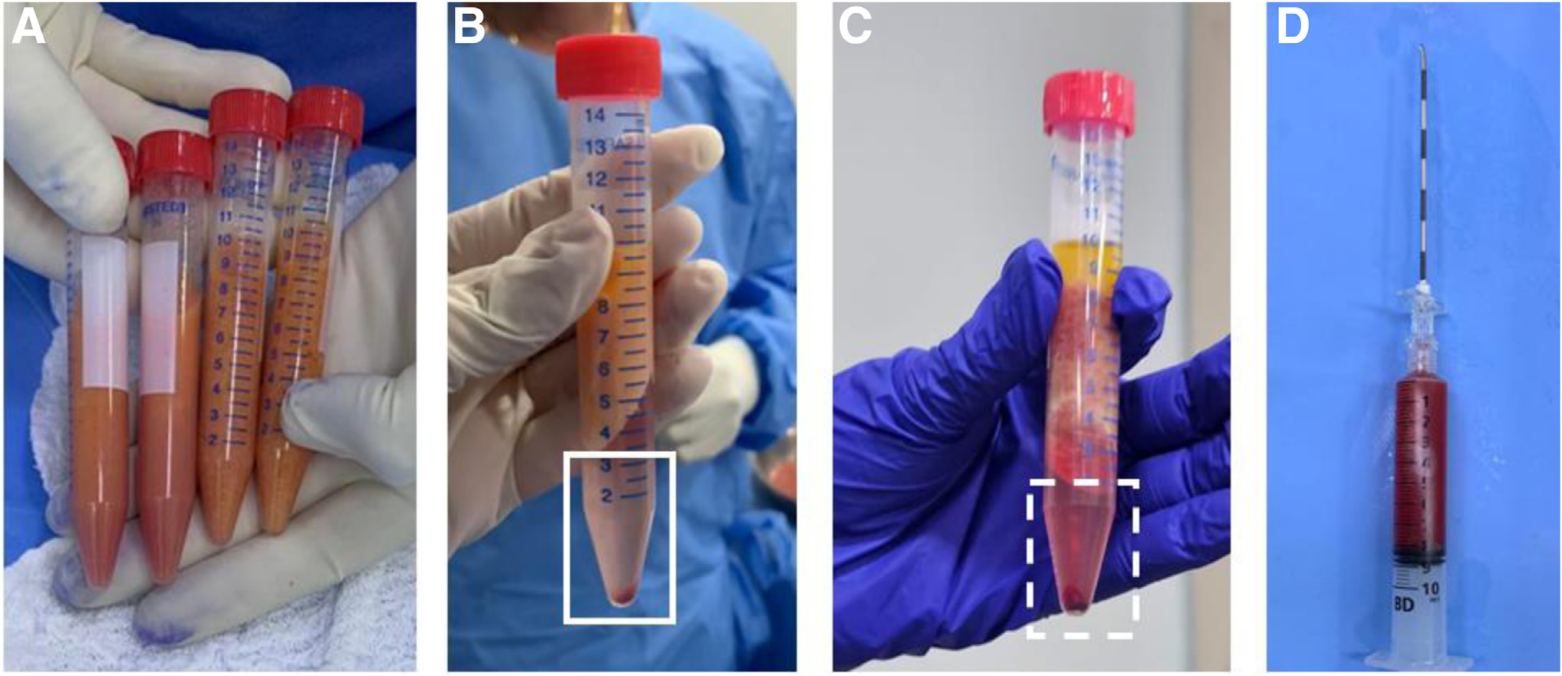
Non-enzymatic stromal vascular fraction isolation protocol. (A) Four Falcon tubes containing the subcutaneous sample are centrifuged for the first stage of processing. (B) The infranatant is aspirated from the tubes and discarded (rectangle). (C) The remaining volume in each tube is filled with saline solution, and the tubes are centrifuged in the second stage. (D) The pellet (infranatant) containing the photo-stimulated stromal vascular fraction (dashed rectangle) is aspirated into a single syringe and is ready for infiltration.

For the non-enzymatic SVF isolation technique, the aspirated tissue is divided into four 10-mL Falcon tubes (Corning, AZ) (Fig 5A) for the first centrifugation, performed at 800*g* for 5 minutes (Fig 5B). After that, the infranatant solution is discarded from the tubes, which are filled with saline solution for the second centrifugation, performed at 700*g* for 5 minutes (Fig 5C). In the end, the pellet (infranatant) containing the photo-stimulated SVF is collected from each Falcon tube in a single syringe and is ready for infiltration (Fig 5D).

After harvesting of the thigh SVF, the patient is prepared for knee arthroscopy, and the limb to be operated on is exsanguinated and kept in an ischemic state by a pneumatic tourniquet. Then, placement of the anterolateral and anteromedial portals is performed. The procedure begins with an articular debridement, in which degenerative meniscal tears and cartilage fragments are removed.

**Fig 6:**
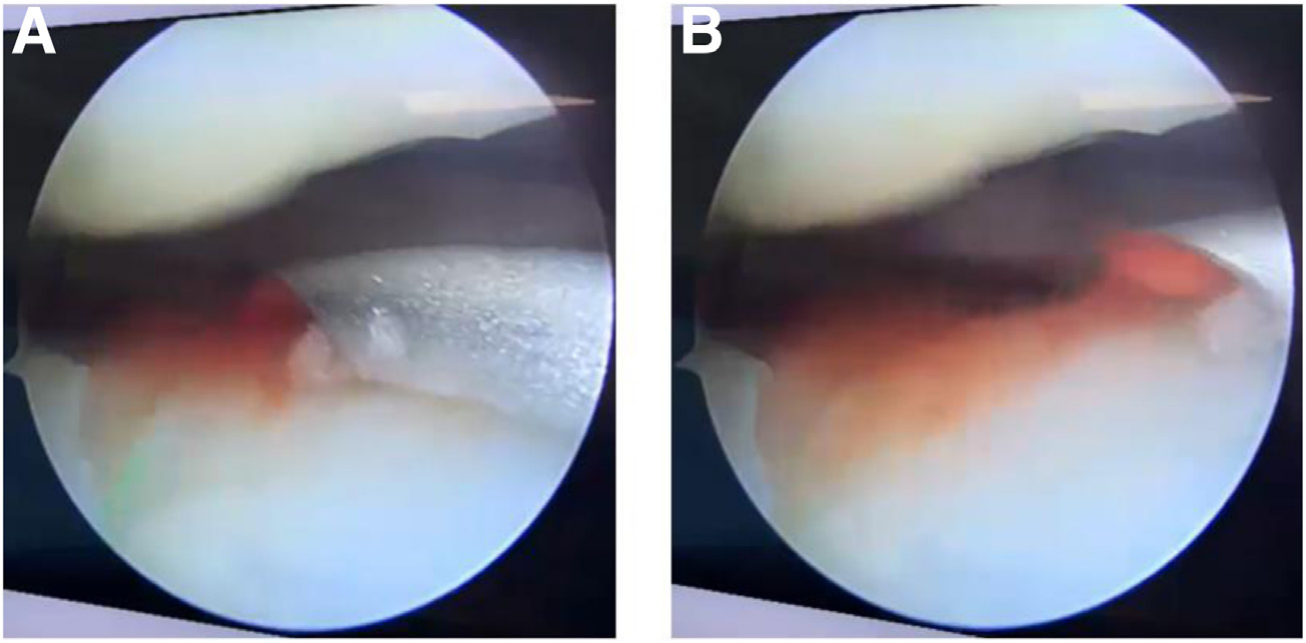
Arthroscopic injection of photo-stimulated stromal vascular fraction. During arthroscopy, the camera is positioned in the anteromedial portal and the needle enters through the anterolateral portal for injection of the autologous stromal vascular fraction graft into the joint (A, B).

During arthroscopy, the autologous SVF graft is prepared by a member of the surgical team. The non-enzymatic isolation protocol as part of the One STEP technique provides 10 mL of photo-stimulated SVF in about 15 minutes in the same operating room. A single-use spinal anesthesia needle attached to a syringe is used to inject the SVF into the knee joint (Fig 6). Wound closure is performed with simple sutures and sterile dressings. Patients are allowed partial weight bearing with 1 crutch on the contralateral side, as tolerated according to pain threshold, for 2 weeks, until the first postoperative consultation is performed. After that, full weight bearing is allowed.

## Discussion

The arthroscopic treatment of mild to moderate KOA using joint debridement associated with an injection of SVF obtained with the One STEP technique is reported in this technical note. The One STEP technique is a non-enzymatic, non-mechanical process avoiding maneuvers such as fat micro-fragmentation, emulsification, filtering, and decanting. The entire photochemical process is performed directly in the subcutaneous tissue in 1 step.^18^^,^^19^ Although SVF has been reported in the treatment of articular cartilage defects and OA,^15^, ^16^, ^17^ most studies have described the use of mechanical or enzymatic processes to obtain it from the adipose tissue.^20^, ^21^, ^22^, ^23^, ^24^

Adipose-derived stem cells (ADSCs) are isolated as part of the aqueous fraction of the lipoaspirate, which is known as the SVF. SVF and ADSCs are similar in terms of properties such as immunomodulation, anti-inflammatory, and angiogenesis properties, although SVF can be acquired without the need for any cell separation or culturing conditions.^25^ The preliminary findings of a study that included 30 patients who received an autologous percutaneous fat injection containing ADSCs indicated that it represents a valid treatment option for KOA.^26^ A meta-analysis of ADSC therapy established the safety and efficacy of both ADSC and SVF therapy for KOA in elderly adults, besides reducing pain and improving knee function.^27^ In 16 patients with bilateral symptomatic Kellgren-Lawrence grade 2 or 3 KOA treated with 4 mL of SVF in 1 knee and 4 mL of hyaluronic acid in the contralateral knee, SVF was considered safe, effectively relieving pain, improving function, and repairing cartilage defects.^15^ A recent study concluded that arthroscopic SVF implantation was useful for repairing cartilage lesions in patients with KOA based on the results regarding pain improvement and cartilage regeneration and the significant correlation between pain and magnetic resonance imaging outcomes at 12-month follow-up.^28^

A systematic review of the literature suggested that although many high-quality studies have been published, owing to the heterogeneity of procedures and adipose tissue derivatives used, as well as the lack of methodologic quality in most studies regarding SVF and ADSCs, their application in the treatment of KOA is not supported.^29^ On the contrary, SVF was considered a safe treatment for KOA and deemed promising in terms of pain, functionality, and anatomic structure improvement, despite the need to standardize the products, homogenize the number of cells, and reduce the use of concomitant treatments to establish proper comparisons.^30^^,^^31^

In this technical note, 40 mL of autologous adipose tissue was lipoaspirate and was processed to obtain 10 mL of SVF. It has been described that the 1,210-nm wavelength has absorption affinity for lipid-rich tissue and stimulates adipocytes and MSCs of the subcutaneous tissue, using the concept of “selective photothermostimulation.”^32^ Hence, besides helping in obtaining the SVF, the laser activates the MSCs, enhancing their phenotype quality^18^ and, consequently, their regenerative potential.^19^

A meta-analysis of 24 clinical trials investigating the use of intra-articular SVF knee injection reported excellent clinical and radiographic results, motivating the standardization of recently developed therapeutic protocols for KOA.^33^ Previous studies only reported SVF harvesting from the abdominal wall^24^^,^^26^ or from the gluteus.^21^

One advantage of the described technique is that the orthopaedic surgeon is more familiar with the thigh anatomy, making the procedure easier than if performed in the abdominal wall, buttock, or flank and, thus, minimizing the risk of adverse events. Hence, all steps of this technique—from adipose-derived SVF liposuction to arthroscopic injection—can easily be performed by the orthopaedic surgeon. Besides, the technique is considered a non-enzymatic method for isolation of the SVF, which is approved for use by regulatory agencies because it is considered a minimal-handling product. However, caution is required during the procedure to avoid invading the deeper layers of the thigh owing to the risk of femoral vessel lesions. Moreover, although this technique is widely used in plastic surgery, its use in orthopaedic surgery is relatively new, requiring additional clinical investigation. Table 2 shows the advantages and limitations of the described technique.

**Table 2:** Advantages, Disadvantages, and Risks

Advantages	Disadvantages	Risks
Laser-assisted subcutaneous harvesting not only helps in obtaining the SVF but also photo-activates the MSCs, enhancing their phenotype quality and regenerative potential.	Specific equipment is required to perform the described technique. A learning curve is necessary.	If the laser catheter or the cannula is moved outside the surgical layer, it can lead to femoral vessel damage.
After subcutaneous harvesting, the laser catheter can be used to reduce dead-space formation, which could lead to a seroma.	Although the technique is widely used in plastic surgery, its use in orthopaedic surgery is relatively new, requiring additional clinical investigation.	If the laser is not properly applied to dissolve the connective tissue and release the parenchyma and stroma, harvesting becomes more difficult and hematoma and/or seroma formation can occur.
Thigh subcutaneous harvesting is easily performed by the orthopaedic surgeon, presenting a lower risk of adverse events.		
The technique is considered a non-enzymatic method for isolation of the SVF, preserving cells in their native environment, which helps in the retention of cellular functions, in addition to being approved for use by regulatory agencies because it is considered a minimal-handling product.		

MSC, mesenchymal stem cell; SVF, stromal vascular fraction.

In conclusion, knee arthroscopy associated with injection of SVF obtained by the One STEP technique is a simple and feasible procedure, with minimal risk of adverse events. The thigh laser-assisted subcutaneous tissue harvesting can be performed by the orthopaedic surgeon and provides an adequate amount of SVF for the treatment of mild to moderate KOA.

## Disclosures

The authors declare the following financial interests/personal relationships which may be considered as potential competing interests: J.P.G.A.F. reports that financial support was provided by DMC Equipments; reports a consulting or advisory relationship with DMC Equipments; and receives speaking and lecture fees from DMC Equipments. R.d.R.M. reports that financial support was provided by DMC Equipments; reports a consulting or advisory relationship with DMC Equipments; and receives speaking and lecture fees from DMC Equipments. P.C. reports that financial support was provided by DMC Equipments; reports a consulting or advisory relationship with DMC Equipments; and receives speaking and lecture fees from DMC Equipments. E.B.d.S. reports that article publishing charges were provided by DMC Equipments.

## Supplementary Data

ICMJE author disclosure forms

ICMJE author disclosure forms

ICMJE author disclosure forms

ICMJE author disclosure forms

Video 1 Procedures for arthroscopy using selective tissue engineering photo-stimulation (One STEP) technique for photo-stimulated stromal vascular fraction (SVF) harvesting. After positioning the patient in the supine position, the right thigh was slightly flexed, externally rotated and abducted, with knee in flexion. The procedure begins with the identification of the area for SVF collection. First, the medial epicondyle is identified. Then, 2 lines are drawn in the anterolateral and posteromedial directions representing the anterior and posterior limits for SVF collection. In the first part of the procedure, the laser catheter is inserted in the medial thigh through the same access positioned at the level of the medial epicondyle. Forward and backward movements are gently performed, similar to a fan, to loosen the subcutaneous tissue using the helium-neon red light. For the tissue collection, a liposuction cannula is attached to a 50-mL syringe, which is inserted in the medial thigh through the same access positioned at the level of the medial epicondyle. Forward and backward movements are performed while pulling back the syringe plunger and using a locking system to maintain the vacuum throughout the harvesting process, obtaining about 40 mL of the photo-stimulated subcutaneous tissue at the end of the procedure. Falcon tubes containing the subcutaneous sample are centrifuged 2 times to obtain the pellet containing the photo-stimulated SVF, which is aspirated into a single syringe and is ready for infiltration. Finally, during arthroscopy, the camera is positioned in the anteromedial portal and a needle is inserted through the anterolateral portal for injection of the autologous SVF graft into the joint.
