# Supplementary material for: Arthroscopy With Adipose-Derived Stromal Vascular Fraction Using a Selective Tissue Engineering Photo-Stimulation Technique for the Treatment of Mild to Moderate Knee Osteoarthritis
Source: Arthrosc Tech. 2024 May 4;13(8):103015. doi: 10.1016/j.eats.2024.103015 (PMC11369935; doi:10.1016/j.eats.2024.103015)
Supplement: ICMJE author disclosure forms [file mmc1.docx]

**Declaration of interests**
 
☐ The authors declare that they have no known competing financial interests or personal relationships that could have appeared to influence the work reported in this paper.
 
☒ The authors declare the following financial interests/personal relationships which may be considered as potential competing interests:

| Patricio Centurion reports financial support was provided by DMC Equipments. Patricio Centurion reports a relationship with DMC Equipments that includes: consulting or advisory and speaking and lecture fees. If there are other authors, they declare that they have no known competing financial interests or personal relationships that could have appeared to influence the work reported in this paper. |
| --- |
